# Modified impact of emotion on temporal discrimination in a transgenic rat model of Huntington disease

**DOI:** 10.3389/fnbeh.2013.00130

**Published:** 2013-09-26

**Authors:** Alexis Faure, Mouna Es-seddiqi, Bruce L. Brown, Hoa P. Nguyen, Olaf Riess, Stephan von Hörsten, Pascale Le Blanc, Nathalie Desvignes, Bruno Bozon, Nicole El Massioui, Valérie Doyère

**Affiliations:** ^1^Centre de Neurosciences Paris-Sud, Université Paris-Sud, UMR 8195Orsay, France; ^2^Centre National de la Recherche ScientifiqueOrsay, France; ^3^Department of Psychology, Queens College and the Graduate Center, CunyNY, USA; ^4^Department of Medical Genetics, University of TuebingenTuebingen, Germany; ^5^Experimental Therapy, Franz Penzoldt Center, Friedrich-Alexander UniversityErlangen-Nürnberg, Germany

**Keywords:** Huntington's disease, emotion, temporal perception, tgHD rats, bisection

## Abstract

Huntington's disease (HD) is characterized by triad of motor, cognitive, and emotional symptoms along with neuropathology in fronto-striatal circuit and limbic system including amygdala. Emotional alterations, which have a negative impact on patient well-being, represent some of the earliest symptoms of HD and might be related to the onset of the neurodegenerative process. In the transgenic rat model (tgHD rats), evidence suggest emotional alterations at the symptomatic stage along with neuropathology of the central nucleus of amygdala (CE). Studies in humans and animals demonstrate that emotion can modulate time perception. The impact of emotion on time perception has never been tested in HD, nor is it known if that impact could be part of the presymptomatic emotional phenotype of the pathology. The aim of this paper was to characterize the effect of emotion on temporal discrimination in presymptomatic tgHD animals. In the first experiment, we characterized the acute effect of an emotion (fear) conditioned stimulus on temporal discrimination using a bisection procedure, and tested its dependency upon an intact central amygdala. The second experiment was aimed at comparing presymptomatic homozygous transgenic animals at 7-months of age and their wild-type littermates (WT) in their performance on the modulation of temporal discrimination by emotion. Our principal findings show that (1) a fear cue produces a short-lived decrease of temporal precision after its termination, and (2) animals with medial CE lesion and presymptomatic tgHD animals demonstrate an alteration of this emotion-evoked temporal distortion. The results contribute to our knowledge about the presymptomatic phenotype of this HD rat model, showing susceptibility to emotion that may be related to dysfunction of the central nucleus of amygdala.

## Introduction

Huntington's disease (HD) is an autosomal dominant neurodegenerative disorder due to an extension of CAG repeat in the coding region of the Huntingtin gene. This pathology is characterized by a triad of psychiatric, motor, and cognitive symptoms along with atrophy of the caudate and putamen, and neuropathological alteration of limbic structures including amygdala and ventral striatum (Bots and Bruyn, 1981; de la Monte et al., 1988; Rosas et al., 2003; Douaud et al., 2006). One important issue in this pathology is the characterization of the presymptomatic stage before clinical motor symptoms appear and the caudate putamen atrophies. In human patients, an affect disorder might be a sign of brain functional alterations preceding neuronal degeneration (Paulsen et al., 2001; Duff et al., 2007; Paradiso et al., 2008; Novak et al., 2012). Among the earliest symptoms, affective processing has been extensively investigated in HD. Deficits in recognition of negative and positive facial expressions, as well as the detection of the expression of anger, disgust and fear are often reported in presymptomatic HD (Duff et al., 2007; Paradiso et al., 2008; Novak et al., 2012; e.g., Henley et al., 2012). Accordingly, in the transgenic rat model (tgHD rats), which exhibits a late adult HD phenotype (von Hörsten et al., 2003), a drastic reduction of anxiety is seen at early stages (von Hörsten et al., 2003; Nguyen et al., 2006; Bode et al., 2008; Urbach et al., 2010). In the same tgHD rat model, we also demonstrated altered emotional and motivational processing at the symptomatic stage (Faure et al., 2011). Emotional alterations might be related to amygdala dysfunction in relation to polyglutamine (polyQ)-containing inclusions and aggregates in central nucleus of amygdala (CE) and shrinkage of this nucleus in these tgHD animals (Petrasch-Parwez et al., 2007; Faure et al., 2011).

In humans, emotion can modulate executive function such as the ability to process time (Droit-Volet and Meck, 2007; Noulhiane et al., 2007; Droit-Volet et al., 2010; Grommet et al., 2011; Gil and Droit-Volet, 2012), although the effects may differ depending upon the task used to assess temporal processing (Gil and Droit-Volet, 2011). What mechanism underlies the effect of emotion on time perception is not clear. Effects have been interpreted in terms of an information processing model with impacts on a putative internal clock consisting of clock, memory and decision stages (Gibbon et al., 1984). Two classes of effects have been put forward. The first links the effect to arousal due to emotion stimuli that leads to an increased speed of the pacemaker of the clock, and overestimation of duration (Angrilli et al., 1997; Droit-Volet et al., 2004; Noulhiane et al., 2007). The second hypothesis supposes that attentional resources are automatically devoted to emotion stimuli at the expense of the timing system leading to an alteration in switch closure, lower levels of pulse accumulation, and consequent underestimation of duration (Droit-Volet and Meck, 2007; Lui et al., 2011). Moreover, a recent study, using a temporal reproduction task, demonstrated that timing precision could also be reduced by the emotional content of the to-be-timed stimulus (Lambrechts et al., 2011). The majority of these studies on emotion-related time distortion have assessed it through the timing of emotionally charged cues. In contrast, only very few studies have looked at the impact of emotion on the timing of neutral cues. In humans, it has recently been shown that fear-inducing films produce, as an aftereffect for up to 5 min, an overestimation of duration with no change in temporal sensitivity (constant Weber ratio), as assessed with a bisection procedure (Droit-Volet et al., 2011).

In animals, the few studies that have addressed the impact of emotion on temporal behavior have yielded different outcomes. One study reported duration overestimation with poorer temporal sensitivity [higher difference limen (DL) and Weber ratio] when bisection tests were performed under stressful conditions (continuous delivery of mild foot-shocks; Meck, 1983), an effect interpreted in terms of an increase in clock speed. Using a peak interval (PI) paradigm, several studies showed that the presentation of an emotion cue, especially with a negative valence through its association with foot-shock, produces a drastic disruption of the temporal behavior when intruded during the to-be-timed stimulus, with a shift to the right of the PI function, often beyond clock reset (Aum et al., 2004, 2007; Brown et al., 2007; Meck and Macdonald, 2007; Matthews et al., 2012), an effect reduced by lesion of the amygdala (Meck and Macdonald, 2007). These underestimation effects could be accounted for by attentional or working memory mechanisms altered by emotion. Interestingly, using the same PI task, Brown et al. (2007) isolated a post-cue effect by presenting the emotion (fear) cue a few seconds before the to-be-timed stimulus. This post-cue effect produced a shift to the right along with a broadening of the PI function.

The affective impairments in presymptomatic HD may have an impact on executive function, as part of the pathology that develops early in this disease. The effect of emotion on temporal behavior has never been tested in human's patients or animal models of HD. A few studies have reported alteration in temporal behavior in HD patients and animal models. In humans, alteration of timing variability has been reported in presymptomatic HD patients in a motor timing task (Hinton et al., 2007). In the transgenic R6/2 mice model of HD, Balci et al. (2009) reported disrupted temporal control in a PI procedure, with intact temporal accuracy, but flatter functions that imply increased variability. Using a bisection procedure in the tgHD rat model, we observed an initial disruption of time perception with poorer sensitivity (Höhn et al., 2011), an effect which disappeared with the repetition of bisection tests (Brown et al., 2011). The bisection procedure, an estimation task, may be more suitable than production tasks (e.g., PI) to test the impact of emotion cues on temporal perception in tgHD animals, as it is less affected by motor or inhibitory control of behavior. In addition, in the PI procedure, the emotionally-valenced distractors presented during or before the to-be-timed stimulus were observed to have effects extending throughout the entire PI trial (e.g., 90 s in duration), a result that is not consistent with the isolation of fast-acting, temporary effects. As the time course of the impact of emotion may be one critical parameter in its interaction with executive functions, we focused on the aftereffects of an emotion cue. We thus chose to use a bisection procedure during which we would be able to examine the acute post-cue effect of emotion on performance by presenting a conditioned emotion (fear) stimulus just before the to-be-timed stimulus.

The temporal bisection procedure in animal research (e.g., Stubbs, 1968; Church and Deluty, 1977) is based on classical psychophysical methodology. During initial discrimination training sessions, the subject is presented on each trial with one of two anchor stimulus durations, short (*S*) or long (*L*) typically signaled by a visual or auditory cue. One response (e.g., left lever press) is rewarded following *S*, while a different response (right lever press) is rewarded following *L*. During subsequent bisection test sessions, additional trials are presented with test durations intermediate between *S* and *L*, with reinforcement omitted. When the probability of a response to the “long” lever [*p*(long)] is plotted against stimulus duration, the resulting function is sigmoidal in shape, resembling the classical psychometric function in the method of constant stimuli (MCS). From that function, the point of subjective equality (PSE) may be obtained by estimating the stimulus value corresponding to *p*(long) = 0.50, and DL may be obtained by estimating the stimulus values corresponding to *p*(long) = 0.25 and 0.75, as in MCS. In animals, the PSE typically falls at the geometric mean of the anchor durations (e.g., Church and Deluty, 1977), but is modifiable by other factors, including intermediate durations (e.g., Raslear, 1983). The Weber fraction defined as DL/PSE provides a measure that is inversely related to temporal sensitivity. The pseudologistic model (PLM, Killeen et al., 1997) is based on a signal detection approach to bisection data, and provides estimates of PSE and gamma, a measure proportional to the Weber fraction. A modified version of PLM (Allan, 2002; Callu et al., 2007; Brown et al., 2011; Höhn et al., 2011) has provided excellent fits to bisection data from human and animal research in terms of proportion of variance accounted for by the model, and was used in the present study to estimate both parameters.

The global aim of the present study was thus to characterize the modulation of temporal discrimination by emotion as a potential marker of presymptomatic symptoms in tgHD animals. As it has never been explored, we first characterized the acute post-cue effect of an emotion (fear) conditioned stimulus on temporal discrimination using a bisection procedure. In a comparative perspective with HD, in which a shrinkage of the central nucleus of the amygdala has been reported (Petrasch-Parwez et al., 2007; Faure et al., 2011), and given the known involvement of this structure in emotional processing and in control or expression of fear responses (LeDoux, 2003; Pare and Duvarci, 2012), we tested the effect of a lesion of the central nucleus of amygdala on the modulation of time processing by a fear cue. We focused on the medial division of the CE as it is the main output of the amygdala responsible for the symptoms of phasic fear (induced by short, discrete cues that are predictably paired with an aversive event). In a second experiment, the protocol was adapted to evaluate the temporal dynamics of the modulation of temporal discrimination by emotion in 7-month old presymptomatic tgHD animals.

## Materials and methods

Here we report two independent series of experiments. Experiment 1 assessed the effect of an emotion cue on temporal discrimination and its dependency upon an intact central amygdala. Experiment 2 aimed at comparing homozygous transgenic animals at a presymptomatic age and their wild-type (WT) littermates in their reaction to emotion cues and related impact on temporal discrimination.

### Experiment 1: effect of CE lesion on emotion-triggered temporal distortion

#### Animals

Twelve male Sprague-Dawley rats (Charles River, Lyon, France) were used in this experiment. Before the present study, these animals had been used in another behavioral study [instrumental discrimination task in other Skinner boxes (Campden instruments) in another experimental room using food reinforcement and a 10-s flashing white light stimulus as the discriminative stimulus].

A 12-h light/dark cycle was maintained during the experiment (light on at 8:00 A.M.). On arrival in the laboratory, rats were given access to food and water *ad libitum* for 2 weeks and handled on a daily basis. All experiments were performed in accordance with the recommendations of the European Economic Community (86/609/EEC) and the French National Committee (87/848) for care and use of laboratory animals.

Daily food amounts were progressively reduced and rats were fed a daily ration to maintain them at 85% of their normal free-feeding weight. The training procedure and apparatus were identical to the one already published (Brown et al., 2011; Höhn et al., 2011), except when otherwise stated.

#### Surgery and histological verification

Three months before the start of the behavioral phase of the present experiment, rats were randomly assigned to two groups. Sham lesioned rats (*n* = 7) and CE lesioned rats (*n* = 5) were anaesthetized with pentobarbital (Sanofi, Libourne, France; 50 mg/kg) and received 0.1 ml injection of atropine (0.25 mg/ml, i.m, Laboratoire Aguettant, Lyon, France) to prevent respiratory problems. They were then placed in a stereotaxic frame, on a thermal barrier to maintain their body temperature (37–38°C). Ibotenic acid (0.2 μl, 10 μg/μl) was injected through a glass micropipette (internal tip diameter: 70–80 hμm) glued to the needle of a 10-μl Hamilton syringe filled with liquid paraffin solution at a rate of 0.1 μl/min in the central nucleus of the amygdala (CE) at the following sites: AP: ±2.56, ML: ±3.9, DV: −7 relative to bregma (Paxinos and Watson, 1986). Glass micropipettes were left in place for 5 additional minutes. After surgery, animals were given an injection of Valium (0.1 ml, i.p., 0.2%, Roche, Neuilly-sur-Seine, France) to prevent seizure activity. Sham animals were subjected to the same treatment except that the micropipette was not introduced in the brain. After data collection, rats were killed with an overdose of pentobarbital (120 mg/kg, i.p.; Sanofi, Libourne, France) and perfused transcardiacally with 100 ml of 0.9% sodium chloride containing 0.5% heparin and 1% sodium nitrite, followed by 300 ml of 4% paraformaldehyde (4°C) in 0.1 M phosphate buffer (PB). Brains were removed, post-fixed for 4 h at 4°C in the same fixative, and immersed in a graded series of sucrose phosphate-buffered solutions (12, 16, and 18%). Serial coronal sections (40 μm thick) were cut on a freezing microtome and collected in an anatomical series. Sections were stained with a Nissl coloration to identify immunostained structures and extension of the lesion.

#### Apparatus

Four operant Skinner boxes (31 × 25 × 31 cm) in sound proof ventilated chambers (background noise 65 dB) were controlled with a GraphicState program (Coulbourn Instruments, Harvard Apparatus, USA). Each chamber was equipped with two left and right metal walls with transparent back panel and front door. A pellet dispenser for delivery of 45 mg grain-based precision pellets in a food cup and two 4-cm retractable response levers were located on the left panel. On the opposite side of the box were a speaker permitting delivery of an auditory stimulus (1 kHz, 80 dB), a red house light illuminated at the beginning of the session to serve as a house light (4 lux), and a green light (25–30 lux) that could be illuminated as a fear cue.

Two chambers (26 × 26 × 50 cm) used for off-the-baseline fear conditioning were located in another adjacent experimental room. Each chamber was equipped with two beige walls, a third (right) beige wall with black vertical strips, and a transparent front door. The grid floor and the green light stimulus on the right panel were identical to the ones used in the operant chamber. A camera connected to a TV monitor allowed remote observation of the behavior of the animal in an adjacent room and storage on videotape for off-line analysis. All experimental events were controlled by custom software running on a PC.

#### Behavioral procedure

***Pretraining.*** Pretraining included one session of magazine training in which 30 pellets were delivered with a mean intertrial interval (ITI) of 60 s (variable intervals ranging between 20 and 100 s). The next 2 days, rats were trained under a continuous reinforcement schedule for each lever separately until 50 reinforcements were earned.

***Temporal discrimination training.*** Reponses to one of two levers (left vs. right) were reinforced following one of two tone durations (2 vs. 8 s). Two blocks of 40 trials, for a total of 80 trials, were presented with equal probability for both tone durations in each session. The relation of tone duration and reinforced response location was counterbalanced between groups. The levers were retracted immediately after a response or after 5 s. The ITI was 30 s on average (range 20–40 s). Rats underwent this training phase until they performed with at least 75% of correct responses for three consecutive sessions. Then, the anchor durations were modified to 2.41 and 6.65 s in order to make the discrimination more difficult and reduce discriminability among stimulus durations during the subsequent bisection tests. This phase was run until the rats reached a criterion of 85% of correct responses.

***Bisection tests.*** Rats were then tested in a psychophysical choice procedure with 5 intermediate durations in a geometric progression (2.85, 3.38, 4, 4.74, and 5.61 s) on non-reinforced trials (12 trials for each duration), in addition to the two training anchor durations (2.41 and 6.65 s, 60 trials each) with reinforcement available. The mean ITI was 30 s. Four bisection sessions were run.

***Test of a fear cue in temporal bisection.*** The fear conditioning procedure was adapted from the one reported previously (Brown et al., 2007). Rats were first submitted to one 40-min session of habituation to the conditioning box. The next day, they were fear conditioned with 10 CS-US pairings in a 44-min session, with a 6-s green non-flashing light immediately followed by a 0.5 s footshock (0.8 mA), and a variable ITI between 2 and 8 min. The next day, rats were placed in the operant chambers to test the impact of the fear cue on the bisection function. For this purpose, bisection tests were run, but for 6 trials of each duration (including reinforced anchor durations), the timing tone cue was preceded by the 6-s green light fear cue, with an onset-to-onset interval of 7 s. It thus defined two types of trials: trials immediately preceded by the CS (Fear-CS condition) and control trials, not immediately preceded by the CS (No-fear-CS condition). This cycle (fear conditioning session, bisection tests) was repeated three times for the next 6 days.

#### Data analysis

Analyses of performances during bisection tests were performed as previously reported (Brown et al., 2011). In brief, response location and latency were recorded for each trial. Bisection data were calculated as *p*(long), the proportion of responses on the lever assigned as correct for the long duration stimulus on all trials with response. The bisection function was analyzed using Prism software with the modified PLM (Killeen et al., 1997) fit for each rat for the bisection test sessions before conditioning and on the averaged curve for the three bisection test sessions assessing the effect of the fear cue. The fit permitted the estimation of the stimulus value corresponding to *p*(long) = 0.5 (PSE), as well as the temporal sensitivity parameter (gamma), which increases as temporal sensitivity decreases.

Contrast analyses of variance (ANOVAs) using VAR3 statistical software (Rouanet et al., 1990) with an alpha level of 0.05 were used for statistical assessments.

### Experiment 2: effect of emotion-triggered temporal distortion in tgHD rats

This emotion experiment followed a previous experiment published in Höhn et al. (2011) with the same rats which addressed a different question, i.e., temporal sensitivity as a function of genotype in presymptomatic models. In the part published in Höhn et al. (2011), rats were first submitted to a 2 vs. 8 s temporal discrimination task at 4.5 months of age. In order to determine the anchor durations for a personalized version of the bisection protocol, a staircase procedure was implemented at 5.5 months of age. The staircase procedure generated anchor durations for each rat that maintained the same target level of discrimination across rats. Testing on the personalized bisection procedure was conducted at 6.5 months. The entire training procedure and the first two sessions of bisection tests have been described in Höhn et al. (2011), and are therefore not reported here.

In the present paper, we report the follow-up part of the experiment, with the unpublished next three sessions of personalized bisection followed by the study of the impact of emotion on the temporal bisection function.

#### Animals

Male transgenic homozygous rats (tgHD, +/+, *n* = 8), and wild-type littermates (WT, −/−, *n* = 8) obtained from an in-house colony (initially generated with 10 pairs of heterozygous rats) and genotyped in Germany as previously described (Kántor et al., 2006) were used. The transgenic HD (tgHD) rat carries a truncated huntingtin cDNA fragment with 51 CAG repeats under the control of the rat huntingtin promoter (von Hörsten et al., 2003). At the beginning of the emotion study, rats were 6.5 months old. Animal care procedures and food deprivation were as described in Höhn et al. (2011).

#### Motor test

A wire suspension test was performed when rats were 7 months old in order to detect signs of deficits in muscle tone. Each animal was hung by its front paws on a horizontal iron bar (4 mm diameter) placed 60 cm above a cushioned floor for a maximum of 1 min. Latencies to fall were measured.

#### Apparatus

Six lever boxes identical to the ones used in Experiment 1 with the same stimuli (lights and tone) in soundproofed chambers were used. The orientation of the equipment was reversed (i.e., levers and magazine on the right wall) for two of the chambers. Animal-box assignment was counterbalanced between groups. The two chambers used for off-the-baseline fear conditioning were the same as those in Experiment 1, located in another adjacent experimental room. Two webcams (one in front of each box) connected to a laptop computer using Anymaze software (Stoelting) allowed videotaping for off-line analysis. All experimental events were controlled by custom software running on a PC.

#### Behavioral procedure

***Personalized bisection.*** With the personalization procedure (first § section Experiment 2: Effect of Emotion-Triggered Temporal Distortion in tgHD Rats), we equalized the difficulty of discrimination across animals and genotypes. Mean anchor values of personalized bisection were similar for both groups (short, 3.135 s ± 0.058, long 5.112 ± 0.095 for tgHD; short, 3.164 s ± 0.061, long 5.067 ± 0.100 for WT). A bisection procedure was implemented with 5 intermediate durations (12 trials each), in addition to the two anchor durations (short and long—60 trials of each), comprising 180 trials in total. The intermediate durations were calculated to be equally spaced between the personalized anchor durations along a logarithmic scale. The ITI varied between 10 and 80 s, with a mean of 30 s. Animals were submitted to 3 days of this bisection procedure before the emotion manipulation was conducted. The last bisection test session, run the day before the emotion phase, is taken as a baseline for the present study.

**Figure 1:**
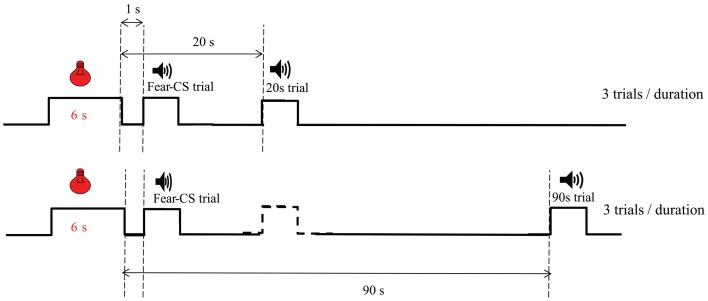
**Diagram of trial types during emotion bisection sessions in Experiment 2**. The temporal arrangement of Fear-CS (1 s), 20 and 90 s types of trials is presented. The dashed line stimulus represents trials which could be present (almost half of the time) between the Fear-CS trials and the 90 s trials.

***Test of a fear cue on personalized temporal bisection (Figure 1).*** The fear conditioning phase was the same as in Experiment 1, except that the fear cue was a 6-s green flashing light (0.5 s ON, 0.5 s OFF), as in Brown et al. (2007). The next day, rats were placed in the operant chambers to test the impact of the fear cue on the bisection function. Four pairs of fear conditioning and personalized bisection test days were run. In bisection tests, for 6 trials of each duration, the timing tone cue was preceded by the 6-s green flashing light fear cue, with an onset-to-onset interval of 7 s (“fear-CS” trials). The other 6 timing tone cue trials per duration were divided into 3 trials on which the tone cue was presented 20 s after the offset of the light fear cue (“20 s” trials), and 3 trials on which the tone cue was presented 90 s after the offset of the light fear cue (“90 s” trials). For the 20 s trials, no trials were inserted between the preceding fear-cue trial and the 20 s trial (Figure 1). For the 90 s trials, on almost half of the trials, they were presented after a 20 s tone trial. The organization of the Fear-CS, 20 and 90 s trials were evenly divided in 3 parts during the bisection session, resulting in the same number of types of trials in each third of the session, i.e., 2 Fear-CS trials, one 20 s and one 90 s trial per duration.

***Non-specific bisection test.*** Following the fear conditioning/bisection cycle, a final day of fear conditioning was performed followed by a final personalized non-specific bisection test day. This bisection test was the same as the one run before the emotion manipulation, i.e., with no presentation of the Fear-CS during the bisection timing procedure. On that day, we intended to test a possible non-specific effect of fear conditioning when given 24 h before bisection, by comparing it with baseline taken before the emotion study.

***Fear cue memory test, reacquisition and extinction.*** One month after the test of the emotional impact of the fear-cue on bisection, animals received a memory test and retraining with the fear conditioning protocol under the same conditions as reported above. Animals were submitted to one session of fear conditioning consisting of three memory trials with the light-CS alone, followed by 10 CS-US pairings. The day after this retraining, animals were submitted to an extinction procedure with 10 CSs delivered without the US.

#### Data analysis

Bisection data were analyzed as in the previous set of behavioral experiments (see above). Contrast ANOVAs using VAR3 statistical software (Rouanet et al., 1990) with an alpha level of 0.05 were used for statistical assessments. Latencies of responses in bisection tests were analyzed from Coulbourn raw data using custom software (Coulbourn Helper v1.1β) developed by Bruno Bozon.

## Results

### Experiment 1: effect of CE lesion on emotion-triggered temporal distortion

Performance during initial temporal discrimination training was similar for Sham and CE rats. All rats reached the first criterion (75% correct responses) in 5–6 training sessions and the 85% criterion within two more sessions. Two rats from the Sham group showed unstable behavior from one day to another during the baseline bisection tests before fear conditioning, and their data were thus eliminated, leaving the study with 5 Sham and 5 CE rats.

#### Assessment of CE lesion

**Figure 2:**
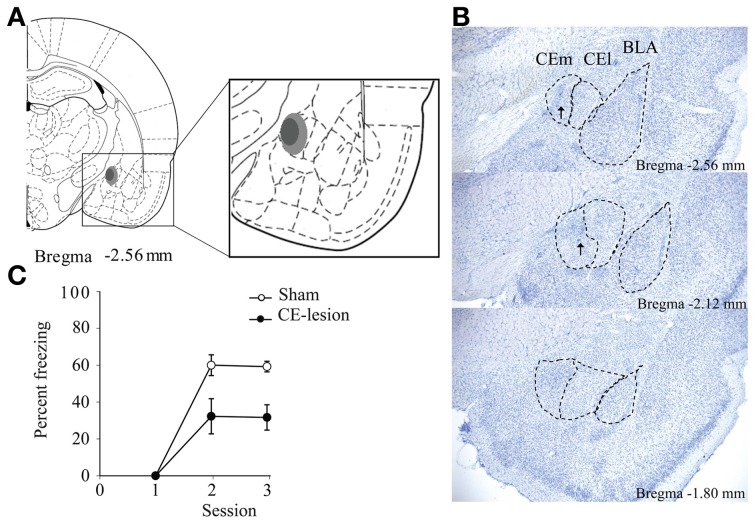
**Bilateral lesion of the medial central nucleus of the amygdala (CE)**. We represent a schematic summarizing the extent of the lesion with the light gray showing the maximum lesion, while the dark gray shows the minimum lesion **(A)**. We also show microphotographs of sections depicting the lesions at three antero-posterior levels in reference to bregma **(B)**. Arrows show the scar due to reactive glial cells. Eventually on **(C)** we represent percent of freezing evoked by the light-CS during the first trial presented during conditioning sessions for sham and CE-lesioned animals. Animals with CE lesion demonstrate significantly less freezing to the light-CS [*F*_(1, 8)_ = 14.35, *p* = 0.005].

All rats showed very similar lesions of the central nucleus of the amygdala, with a nearly complete destruction of the medial part of the nucleus, and a 23% minimum and a 51% maximum destruction of whole structure (Figures 2A,B).

As expected, and in agreement with the literature on the role of CE in the processing and storage of the fear memory trace (Goosens and Maren, 2001; Hinton et al., 2007; Pare and Duvarci, 2012), CE-lesioned animals showed poorer memory of fear conditioning compared to Sham animals, as assessed through the freezing evoked by the light-CS during the first trial presented in the second and the third conditioning sessions (Figure 2C).

#### Bisection tests

**Figure 3:**
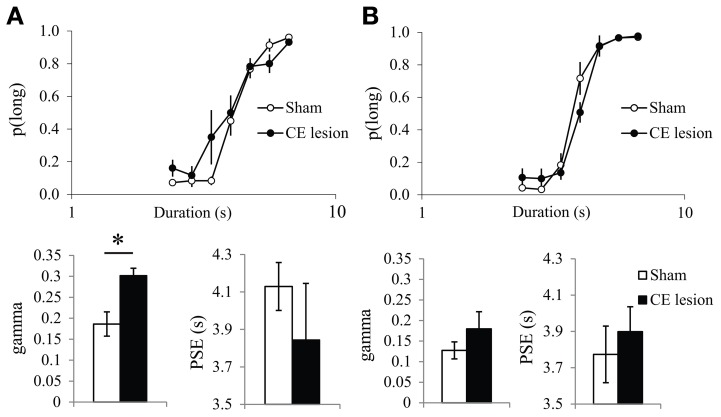
**Bisection curves and temporal parameters extracted from the fitted bisection function (PSE and gamma) for sham (in white) and CE lesioned animals (in black) during the first (A) and the fourth bisection test day (B) before the fear conditioning phase**. ^*^p < 0.05.

Previous work in rats has shown that performance during repeated bisection tests may evolve with repetition, and that effects of a lesion on bisection performance may be transitory (Callu et al., 2009; Brown et al., 2011). Here too, there was a clear evolution of performance with the repetition of bisection tests. Considering the proportion of responses on the lever assigned as correct for the long duration stimulus [*p*(long)] during the first bisection test after discrimination training, CE-lesioned animals showed different temporal performance with a shallower slope compared to Sham animals (Figure 3A). Statistical analyses of *p*(long) as a function of stimulus duration confirmed a significant group × duration interaction [*F*_(6, 48)_ = 2.38, *p* < 0.05], with no group difference (*F* < 1). Parameters from the pseudologistic fits showed no difference in PSE (Sham 4.13 ± 0.13 vs. CE 3.84 ± 0.30, *F* < 1), or the proportion of variance accounted for by the fit [*R*^2^, Sham 0.97 ± 0.01 vs. CE 0.89 ± 0.06; *F*_(1, 8)_ = 1.72, ns], but a significant difference in gamma [Sham 0.19 ± 0.03 vs. CE 0.30 ± 0.02, *F*_(1, 8)_ = 11.48, *p* < 0.01], suggesting disrupted temporal sensitivity in the lesioned group.

With repetition of bisection tests, the differences between the two groups diminished. On the bisection test performed the day before the start of the emotion phase (Figure 3B), the bisection curves still differed significantly [group × duration interaction, *F*_(6, 48)_ = 2.66, *p* < 0.05], but there was no longer a significant difference in gamma [Sham 0.13 ± 0.02 vs. CE 0.18 ± 0.04, *F*_(1, 8)_ = 1.27, ns], and still no difference in PSE [Sham 3.77 ± 0.16 vs. CE 3.90 ± 0.14, *F* < 1] or in *R*^2^ [*F*_(1, 8)_ = 1.44, ns; 0.99 ± 0.004 vs. 0.95 ± 0.04 for Sham and CE groups, respectively]. Finally, no significant differences were detected on a final bisection test performed the day after the last emotion test (group × duration interaction *F* < 1; gamma 0.13 ± 0.03 vs. 0.16 ± 0.03; PSE 3.77 ± 0.16 vs. 3.77 ± 0.18; *R*^2^ 0.97 ± 0.02 vs. 0.99 ± 0.01 for Sham and CE groups, respectively; all *F*s < 1).

Thus, the initial disruption of performance seen in CE animals during bisection tests was alleviated by repetition of the bisection procedure.

#### Emotion-triggered temporal distortion

**Figure 4:**
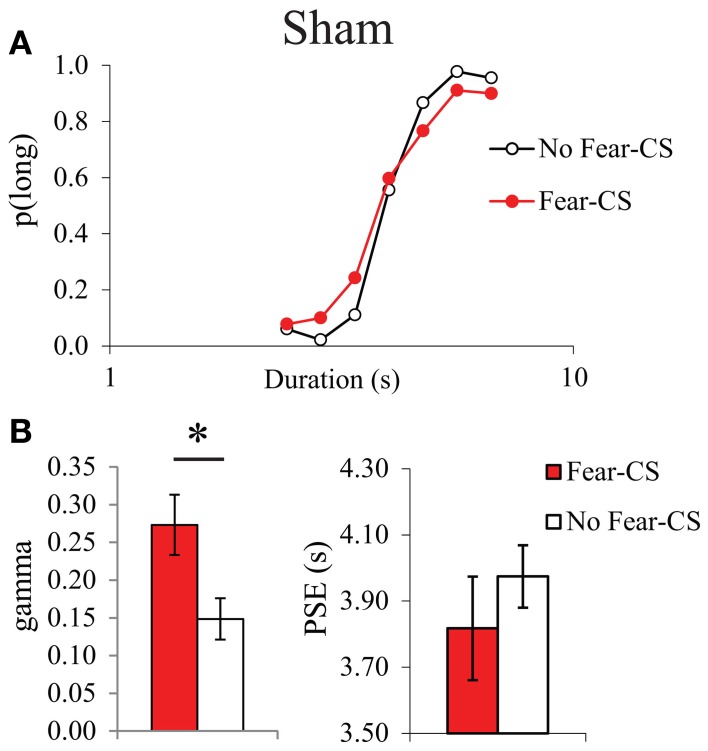
**Bisection curves, averaged over the three testing sessions **(A)**, and temporal parameters extracted from the fitted bisection function (PSE and gamma) **(B)** for Sham animals in trials immediately preceded by a Fear-CS (red) and trials without Fear-CS (No-fear-CS, white)**. ^*^p < 0.05.

As it had never been tested before, we first analyzed the acute post-cue effect of the presentation of a fear cue on temporal bisection performance in Sham animals. The characterization of the effects of the presentation of the fear CS cue on the temporal performance was analyzed through the comparison of the trials immediately preceded by the CS (Fear-CS condition) with the other control trials, not immediately preceded by the CS (No-fear-CS condition), averaged across the three testing sessions. The corresponding bisection curves obtained in Sham animals show a shallower curve in the fear-CS-condition as compared to the control No-fear-CS condition (Figure 4A). A significant duration × condition (Fear-CS vs. No-fear-CS) revealed that the temporal sensitivity was poorer immediately after the presentation of the Light cue trials (Fear-CS condition), as compared to other control trials [No-fear-CS condition; *F*_(6, 24)_ = 2.69, *p* < 0.05], with no significant effect of trial condition on *p*(long) (*F* < 1). This effect was reflected in the estimated parameters from the pseudologistic fits (with all *R*^2^ > 0.89), with a significant increase in gamma on Fear-CS trials [*F*_(1, 4)_ = 7.41, *p* = 0.05], but no significant change in PSE [*F*_(1, 4)_ = 1.87, ns] (Figure 4B). Thus, the fear cue acutely disrupted the temporal sensitivity in Sham animals.

**Figure 5:**
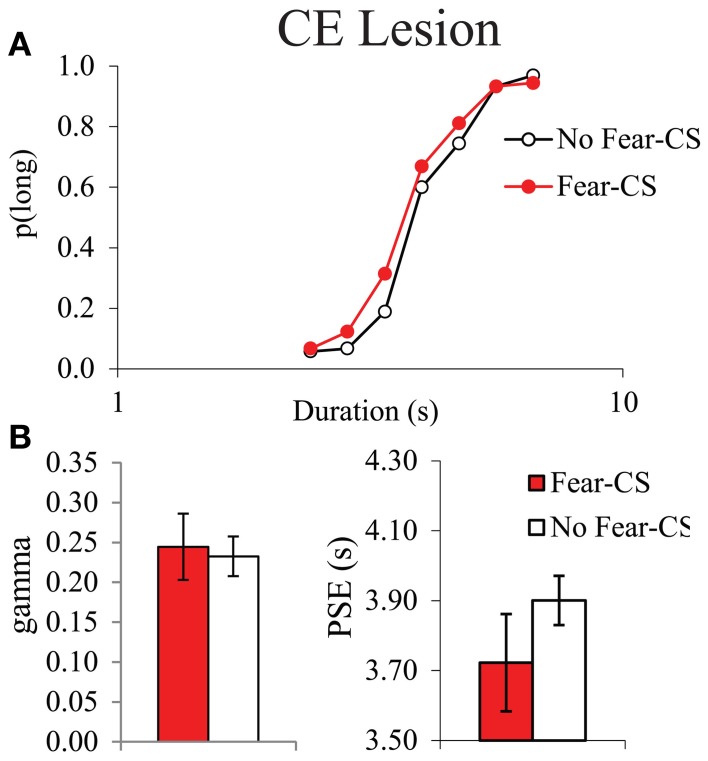
**Bisection curves, averaged over the three testing sessions **(A)**, and temporal parameters extracted from the fitted bisection function (PSE and gamma) **(B)** for CE lesioned animals in trials immediately preceded by a Fear-CS (red) and trials with No-fear-CS (white)**. ^*^p < 0.05.

In contrast, no disruption in temporal sensitivity was observed in the CE group (Figures 5A,B), as no significant duration × condition interaction [*F*_(6, 24)_ = 1.34, ns], and no significant difference in gamma between conditions (*F* < 1) were obtained. There was no significant condition effect for *p*(long) [*F*_(1, 4)_ = 5.98, ns], or for PSE [*F*_(1, 4)_ = 5.72, ns]. During the fear tests, the absence of a difference in gamma on Fear-CS vs. No-fear-CS trials in group CE (Figure 5B) was accompanied by an increase in gamma on both trial types as compared to baseline trials (Figure 3B). While unanticipated, the latter increase may reflect a non-specific disruption of sensitivity owing to fear cue presentations that re-established the sensitivity difference initially observed between groups (Figure 3A). That is, gamma may be a parameter that is sensitive to novel situations, especially in lesioned animals (see, for example, Callu et al., 2009). In any case, there was no evidence of a specific effect of the Fear-CS on gamma in group CE. Thus, the emotion-triggered disruption of temporal discrimination observed in Sham animals was eliminated by the lesion of medial CE.

### Experiment 2: emotion-triggered temporal distortion in presymptomatic tgHD rats

In the previous experiment, despite the reduced difference we had chosen for anchor durations, there was still a tendency for discrimination between reinforced (anchor durations) and non-reinforced (intermediate durations) trials, as detected through a tendency toward longer latencies for the middle durations when bisection tests were repeated, similarly for both Sham and CE groups (data not shown). Therefore, for the study in the transgenic animals, we developed a strategy for testing bisection performance with lower discriminability among testing durations, personalized for each rat. In addition, we designed the present experiment with bisection test trials at definite times (1, 20, and 90 s) after the fear-cue trials (Figure 1) in order to analyze more precisely the dynamics of the temporal distortion induced by the fear cue.

Data of one tgHD animal were discarded in the data analyses (except for motor assessment) because this animal stopped responding during the bisection tests with the fear cue. Analyses were thus performed on 7 tgHD and 8 WT.

#### Motor assessment

As previously shown in tgHD rats (Faure et al., 2011), the wire suspension test showed no genotype difference in drop latencies of 7-months old animals (tgHD 25.54 ± 6.24 s, WT 28.21 ± 7.45 s) [*F*_(1, 14)_ < 1], thus confirming their presymptomatic status.

#### Fear conditioning

**Figure 6:**
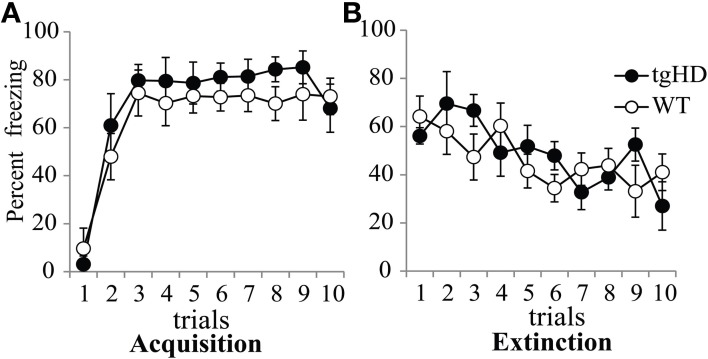
**Percent of freezing evoked by the light-CS during the first 10-trial session of acquisition (A) and the 10-trial session of extinction (B) for tgHD (black circle) and WT animals (open circles)**.

During fear conditioning, both tgHD and WT rats learned a fear response to the CS, to the same extent. During the first conditioning session (Figure 6A), freezing to the CS increased over successive trials [*F*_(9, 117)_ = 29.83, *p* < 0.001] with no genotype effect [*F*_(1, 13)_ = 1.397, ns], nor genotype × trial interaction (*F* < 1). Comparison of the level of freezing between tgHD and WT animals either during the first or the last trial of each of the five conditioning sessions did not show any difference between the two groups in the acquisition or memory of CS-evoked fear (data not shown, *F*s < 1). One month after the end of the experiment, the animals were tested for long-term memory for CS-evoked fear, followed by an extinction session. No significant difference was observed between the two groups during the long-term memory test [*F*_(1, 13)_ = 1.56, ns], nor during extinction (Figure 6B, no group effect, *F* < 1). There was a significant group × trial interaction [*F*_(9, 117)_ = 2.85, *p* < 0.01], but no genotype difference on the first (*F* < 1) or last trial [*F*_(1, 13)_ = 1.71, ns], suggesting no genotype difference in overall extinction learning. Therefore, WT and tgHD rats showed similar fear conditioning acquisition, memory, and extinction.

#### Personalized bisection

***Baseline and non-specific personalized bisection.*** The analysis of proportion of “long” choice during the bisection session (baseline) performed the day before the start of the emotion manipulation showed an effect of duration on *p*(long) [*F*_(6, 78)_ = 143.34, *p* < 0.01], with no effect of genotype (*F* < 1), and no genotype × duration interaction [*F*_(6, 78)_ = 1.16, ns]. Results were similar during the bisection session performed the day after the last conditioning session (test for non-specific effect of fear conditioning) [all *F*s < 1, ns, except for the duration effect *F*_(6, 78)_ = 113.26, *p* < 0.01]. Furthermore, there was no difference between these two sessions, either for the WT group [*F*_(1, 7)_ = 2.24, ns] or for tgHD rats (*F* < 1). During those two personalized bisection sessions, response latencies were not affected by stimulus duration, either for WT [*F*_(6, 42)_ = 1.32, ns] or for tgHD rats [*F*_(6, 36)_ < 1, ns], demonstrating that personalization, which equalized the difficulty of discrimination for each animal, abolished the progressive increase in latency during intermediate durations seen in tgHD animals with repetition of classical bisection testing (Brown et al., 2011).

***Emotion test: personalized bisection with fear cue.*** For the four sessions during which the effect of the fear-CS was assessed, we first determined whether the effect of the fear-CS was changing over the course of the test session, with repeated CS-alone presentations. For this purpose, proportion of “Long” responses restricted to fear-CS trials was compared among three successive trial-blocks comprising the emotion bisection sessions. This analysis revealed a significant effect of trial-block on *p*(long) [*F*_(2, 26)_ = 5.18, *p* < 0.05], with no genotype effect (*F* < 1), and no interaction between genotype and trial-block [*F*_(2, 26)_ = 1.45, ns]. During the ten trials of fear extinction performed in subsequent testing (Figure 6B), the animals showed a significant decrease in freezing behavior to the CS [*F*_(9, 117)_ = 6.66, *p* < 0.001]. As 14 CSs were presented in each of the three trial blocks of the emotion bisection tests, extinction effects could thus be suspected during the course of emotion cue testing, consistent with the within-session effect on *p*(Long) during fear-CS trials. Therefore, we restricted our following analyses to the first trial-block of emotion bisection sessions, during which the effect of the fear-CS was maximal.

**Figure 7:**
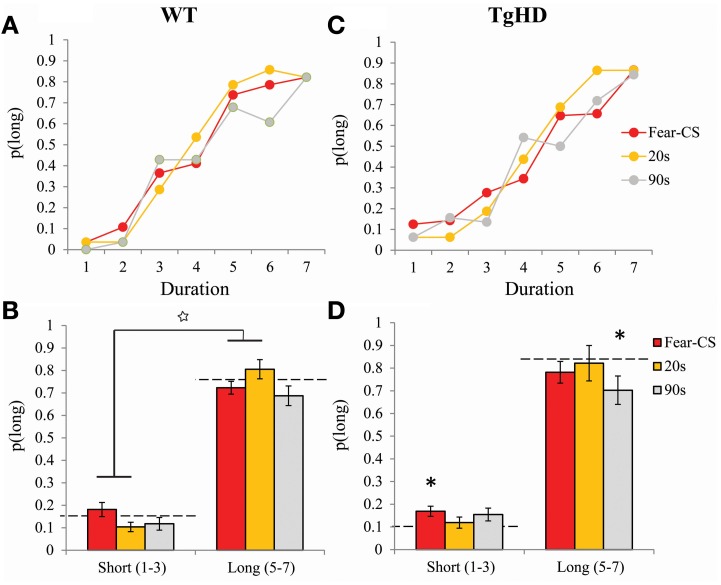
**Bisection curves restricted to the first third of session averaged across the 4 bisection tests with Fear-CS, 20 and 90 s trial types in WT (A) and tgHD (C) animals**. Averages of *p*(long) for long (D5–D7) or short (D1–D3) duration ranges in Fear-CS, 20 and 90 s trials in WT **(B)** and tgHD **(D)** animals. ^*^p < 0.05, difference from baseline (horizontal dashed lines); star means significant duration × trial type interaction, *p* < 0.05.

In the personalized emotion bisection procedure, restriction of analyses to the first third of the session during the 4 days of bisection tests yielded an averaged bisection curve based on 8 choice trials per duration for fear-CS trials and 4 choice trials per duration for 20 and 90 s trials (Figures 7A,C). Animals displayed typical bisection curves with proportion of “long” responses increasing with signal duration [*F*_(6, 78)_ = 138.69, *p* < 0.001]. There were no main effects of genotype or type of trial [*F* < 1 and *F*_(2, 26)_ = 1.23, ns], but these factors modulated the proportion of choice of “long” as evidenced by a significant interaction between stimulus duration and genotype [*F*_(6, 78)_ = 2.64, *p* < 0.05] and an interaction between duration and type of trials [*F*_(12, 156)_ = 2.02, *p* < 0.05]. Therefore, we refined the analyses by comparing mean of proportion of “long” responses separately for short (D1–D3) and long (D5–D7) stimulus duration ranges for each group of animals (Figures 7B,D).

Averaging *p*(long) within long (D5–D7) and short (D1–D3) duration ranges, we obtained a significant interaction between duration range and type of trials for both WT [*F*_(2, 14)_ = 5.8, *p* < 0.02] and tgHD animals [*F*_(2, 12)_ = 4.22, *p* = 0.04]. This result indicates a differential effect of emotion for short and long duration ranges. In particular, in WT animals, an increase in *p*(long) for the long duration range along with a decrease of *p*(long) for the short duration range during 20-s type of trial, as compared to the fear-CS trial, was reflected in a significant interaction between duration range and fear-CS vs. 20 s trial type [*F*_(1, 7)_ = 9.84, *p* < 0.02]. This pattern was not observed between fear-CS and 90 s type of trials (no duration range by trial type interaction, *F* < 1, ns). In tgHD animals, none of these interactions was significant [duration × fear-CS vs. 20 s *F*_(1, 6)_ = 5.02m, *p* = 0.067 and duration × fear-CS vs. 90 s, *F*_(1, 6)_ = 2.32, *p* = 0.18]. When compared to the baseline bisection session that was run prior to fear conditioning (Figures 7B,D, horizontal dashed lines), for short and long ranges none of the values of *p*(long) was different from baseline in WT animals [all *F*s_(1, 7)_ < 3.39]. In tgHD animals, *p*(long) was significantly different from baseline for fear-CS trials in the short range [*F*_(1, 6)_ = 5.938, *p* = 0.05], and for the 90 s trial type in the long range [*F*_(1, 6)_ = 16.36, *p* < 0.01]. All the other comparisons were not significant [*F*s_(1, 6)_ < 3.73, ns].

***Pseudo-logistic fit.*** Presumably due to the personalized version of the bisection tests, some curves were difficult to fit with a good confidence, as revealed by lower variances accounted for (*R*^2^). In order to compare the distribution of the *R*^2^ in baseline bisection, emotion, and non-specific bisection sessions, we took 8 trials per duration during the first two thirds of the baseline and non-specific sessions to compare between these conditions and the 8 trials per duration for fear-CS trial condition and the 4 trials per duration in 20 and 90 s trials of the first third of emotion bisection sessions.

**Figure 8:**
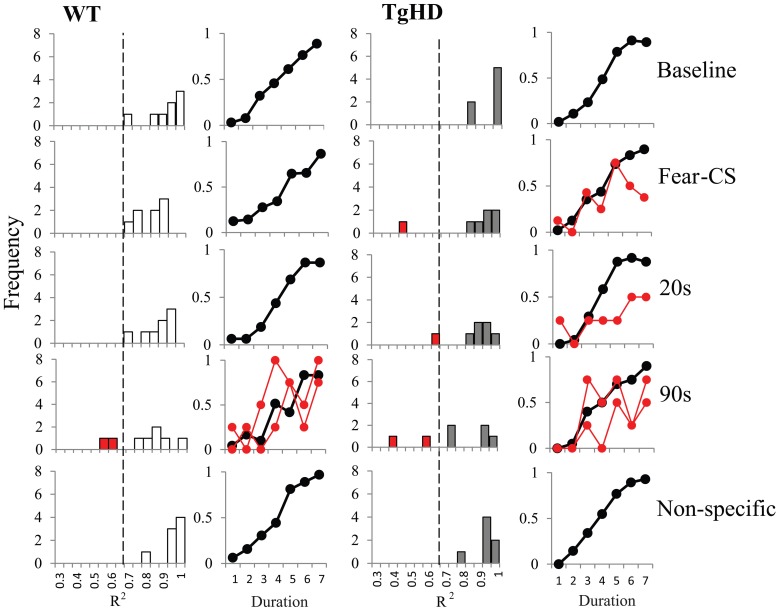
**Distribution of *R*^2^ and averaged bisection curves with *R*^2^ > 0.65 for WT (left panel) and tgHD (right panel) animals during baseline and non-specific sessions and during Fear-CS, 20 and 90 s trials in emotion bisection test sessions (8 trials per duration in all conditions; see text)**. In red we present the individual bisection curves for animals with *R*^2^ under 0.65.

The distributions of the *R*^2^ values for both groups are shown Figure 8. There was a tendency for greater spread of *R*^2^ values on 90 s trials for both genotypes. Analyses of *R*^2^ on fear-CS, 20 and 90 s trial types revealed no genotype (*F* < 1) or type of trial effect [*F*_(2, 26)_ = 2.7, *p* = 0.09].

**Figure 9:**
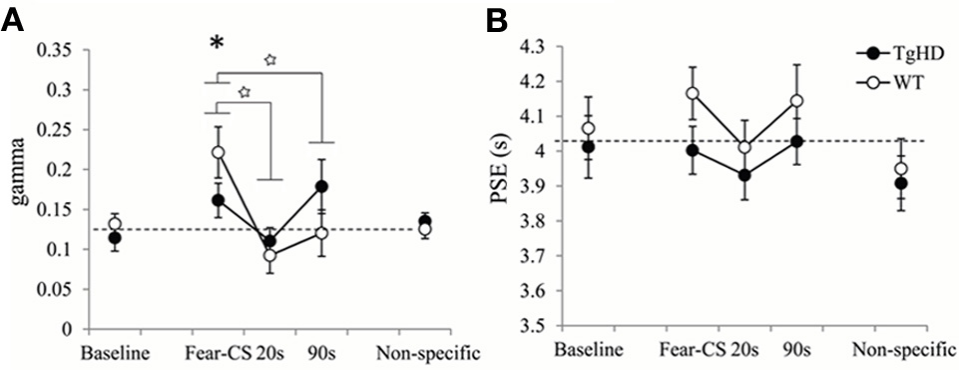
**Temporal parameters extracted from the fitted bisection function averaged across four emotion bisection sessions, with gamma (A) and PSE (B) for WT and tgHD animals in baseline and non-specific sessions, and on Fear-CS, 20 and 90 s trials of emotion sessions**. ^*^p < 0.05, difference from baseline; star means significant group × trial type interaction, *p* < 0.05.

The distribution of *R*^2^ values for WT animals led us to choose a threshold at 0.65 for accepting or rejecting the fit. As shown in Figure 8, some WT and tgHD animals would thus have no fit value of gamma or PSE on certain types of trials. When possible, we replaced the missing value with the value of the fit obtained for the following part of the session. For example if an animal had no good fit on the first part of the bisection session, we used its fit value from the second part. Only one WT and one tgHD animal fell in this category, for the 90 s trial type. However, all data for one tgHD animal were discarded because that animal had below-threshold *R*^2^ values for both fear-CS and 90 s types of trial on the first part of the session. Data for one WT animal were also discarded because this animal never had acceptable *R*^2^ values on the 90 s type of trial fit in any of the part of the session. We thus performed the analyses on 6 tgHD animals and 7 WT animals (Figures 9A,B).

During the session in which the fear-CS was presented, the effect of the fear cue on the temporal precision (gamma) was modulated by time elapsed (1, 20, or 90 s) from the fear cue presentation to the timing signal (Figure 9A). This was true for both WT [effect of trial type *F*_(2, 12)_ = 9.35, *p* < 0.01] and tgHD animals [*F*_(2, 10)_ = 4.83, *p* < 0.05]. Moreover, a significant genotype × trial type interaction [*F*_(2, 22)_ = 4.72, *p* = 0.02] demonstrated that this temporal evolution was different for the two genotypes. When restricted to fear-CS and 20 s trial types, a significant genotype × trial type interaction [*F*_(1, 11)_ = 5.60, *p* < 0.05] indicated that tgHD animals showed a smaller effect of trial type on gamma. However, there was a significant decrease in gamma from fear-CS to 20 s trial type for both WT [*F*_(1, 6)_ = 17.57, *p* < 0.01] and tgHD animals [*F*_(1, 5)_ = 28.22, *p* < 0.01]. When comparing fear-CS and 90 s trial types, there was also a significant genotype × trial type interaction [*F*_(1, 11)_ = 9.08, *p* < 0.02]. The decrease in gamma was significant on 90 s trials compared to fear-CS trials for WT [*F*_(1, 6)_ = 12.04, *p* < 0.02], but not for tgHD animals (*F* < 1, ns), suggesting a return to the baseline level for WT animals and an altered gamma in tgHD animals at 90 s. In comparison to the baseline level, the temporal precision during fear-CS trials was significantly worse (higher gamma) for WT animals [*F*_(1, 6)_ = 7.33, *p* < 0.05], an effect which was not found in tgHD animals [*F*_(1, 5)_ = 3.01, ns]. Gamma values for 20 and 90 s trial types never differed from baseline for WT animals [baseline vs. 20 s *F*_(1, 6)_ = 2.66, ns, and baseline vs. 90 s *F* < 1] or for tgHD animals [baseline vs. 20 s *F* < 1, and baseline vs. 90 s *F*_(1, 5)_ = 3.17, ns].

The results for PSE values are shown in Figure 9B. Although WT rats tended to have longer PSE values than tgHD animals during the emotion tests on bisection, there was no significant genotype effect [*F*_(1, 11)_ = 3.23, ns], nor genotype × trial type interaction (*F* < 1). Moreover, there was no effect of type of emotion trials for WT [*F*_(2, 12)_ = 1.05, ns] or tgHD animals (*F* < 1). In comparison to baseline level, none of the PSE values during emotion trials were found to be significantly different (*F*s < 1).

## Discussion

In the first experiment, we showed in sham animals that a fear cue, when presented just before a to-be-timed stimulus in a temporal bisection task, induces a decrease in temporal precision (increase in gamma). This effect was abolished in animals with medial CE lesion, a lesion which decreased emotional freezing response to the fear CS, thus demonstrating the involvement of this part of the amygdala in the modulation of temporal processing by emotion. In the second experiment, the decrease in temporal precision induced by the fear cue was replicated in WT animals. Our experimental design revealed, however, that this effect was short-lived, as it was observed when the to-be-timed stimulus was presented 1 s after the offset of the fear cue, but not when the to-be-timed stimulus was presented 20 or 90 s later. Our 7-month old presymptomatic tgHD animals exhibited conditioning and extinction of responding to a fear-CS at the same rate as WT animals. However, these tgHD animals showed a different dynamic for the effect of the fear-CS on temporal precision. The analyses of gamma and *p*(long) both suggest an immediate short-lasting effect in WT animals [immediate increase in gamma and group × trial type interaction in *p*(long) between fear-CS and 20 s trial], whereas the effect tended to be smaller at a short delay (Fear-CS trial) but present 90 s later in tgHD rats [type of trial effect on gamma, interaction with genotype, and no interaction between group and fear-CS vs. 90 s trials on *p*(long)], with a temporary return to baseline level at 20 s (Figures 7, 9).

Our experimental design was aimed at testing the dynamic of the after-effect induced by an emotion cue on temporal processing, a question that has not been addressed in previous experiments either in humans or animals. Our main significant finding in both sham animals (Experiment 1) and WT animals (Experiment 2) is an acute deterioration of temporal precision, present at 1 s, but absent by 20 s after the presentation of a fear cue. This effect of emotion on temporal sensitivity has not been reported in human or animal studies testing temporal estimation of emotion stimuli. However, recent data in humans reported a deleterious effect of emotional content on temporal precision which lasted at least 6 s, in a temporal reproduction task (Lambrechts et al., 2011). Furthermore, training rats under constant mild foot shock led to a decrease in sensitivity to time evidenced in a bisection task with an increased DL and Weber ratio (Meck, 1983). In a situation testing the post-cue effect of a fear cue in a PI procedure, we also observed a widening of the PI function compared to control trials, indicating poorer temporal sensitivity (Brown et al., 2007). The fact that the effects could be observed in a PI procedure with an FI 30 s schedule, while disruptive effects were no longer observed after 20 s in the present experiment may be due to methodological factors. Only 12 fear cue trials per session (for 4 sessions) were delivered in the PI study, whereas in the present study, the animals were exposed to 42 fear cue trials per session (for 4 sessions). It is quite likely that extinction processes may have resulted in an underestimation of the time course of the disruptive effects. This may also explain why no systematic effects were observed on PSE. As summarized in the introduction, conflicting results (overestimation and underestimation) have been reported in the literature, and the effects of emotion on temporal behavior may have different time courses depending on the range of the temporal stimuli (Lambrechts et al., 2011), or other factors that remain to be determined. In any case, the present results suggest that gamma and PSE are dissociable.

In sum, our data clearly demonstrate that presentation of a fear-CS induces a robust detrimental effect on temporal discrimination which outlasts its presentation. Possible contributors of this effect maybe non-temporal factors such as attentional or motivational disruption, as emotional and motivational alterations have been found in symptomatic tgHD animals. Alternatively, the effects may be mediated by temporal factors. In the framework of the Scalar Expectancy Theory (Gibbon et al., 1984), the post-cue effect of emotion may have targeted different levels of temporal information processing. At the clock stage, an emotion cue may induce variability in the pacemaker rate or a modified flickering of the switch, both leading to increased variability in pulse accumulation and decreased temporal precision as previously suggested (Lambrechts et al., 2011). Note that modified switch function does not entail systematic differences in mean flicker rate, but in variability in the flicker duty cycle, which would yield increased variability in accumulated subjective time. Interestingly, although not in the timing domain, a recent study has reported that attention produces an increase in neuronal communication with a change in synaptic plasticity in sensory neural networks (Briggs et al., 2013). Extended to the temporal domain, this type of mechanism at the neural level could result in a decreased temporal sensitivity when attention is diverted from the processing of temporal cues, and would thus result in an increased variability in temporal perception. Finally, at the decision stage of the clock, an unstable criterion threshold could also account for an increase in gamma, and thus increased variability in the temporal behavior. The susceptibility of timing performance to the foregoing influences on temporal precision suggests that temporal precision may be a sensitive tool in the assessment of disruption of executive function, in line with the finding that it is readily disrupted in various assays, including post-cue effects (the present data; Brown et al., 2007) and in animal models of pathology (the present data; Callu et al., 2009).

Similar to what has been reported in tgHD rats (Brown et al., 2011; Höhn et al., 2011), medial CE lesioned animals showed an initial disruption (poorer temporal precision) in the temporal bisection task, an effect that disappeared progressively with repetition of the bisection tests (Figure 3). The influence of the medial central nucleus of amygdala on temporal precision could be related to its role in circuits that process attentional responses to salient appetitive stimuli (Han et al., 1997; Holland et al., 2000), suggesting a deficit in attentional orientation to the to-be-timed stimulus, with consequent enhanced clock variability, that is alleviated with increased amounts of training.

The disruption of temporal processing by emotion observed in sham and WT animals was abolished in medial CE lesioned animals and reduced in presymptomatic tgHD rats. Lesions of amygdala (central and basolateral nucleus) have already demonstrated the involvement of this structure in the focalization of selective attention to the emotion stimulus at the expense of timing (Meck and Macdonald, 2007). The lack of influence of the fear-CS on temporal processing in medial CE lesioned animals was not due to a ceiling effect, as the same animals showed poorer temporal sensitivity (more elevated gamma) during the first bisection tests. Rather, it might be due to a deficit in processing the emotional value of the fear-CS as suggested by a reduction of freezing and theories proposing a role of CE in Pavlovian fear learning (Balleine and Killcross, 2006). However, the presymptomatic tgHD animals also showed a reduced impact of the fear-CS on temporal precision without any alteration of the fear response to the CS (as assessed through the freezing measure). Our 7-month old tgHD animals could be considered as presymptomatic, as they showed no motor symptoms in the wire suspension test, a result consistent with previous studies demonstrating that motor alteration in this tgHD model carrying only 51 CAG repeats could appear around 6–9 months, but possibly not before 11 months, and that motor function worsens with age (Cao et al., 2006; Nguyen et al., 2006; Faure et al., 2011). Furthermore, in symptomatic tgHD animals, previous data have demonstrated a normal freezing response which increased as a function of age to levels higher than those for WT animals (Faure et al., 2011). Moreover, recent findings on R6/2 mice demonstrated alteration of fear extinction in relation to prefronto-amygdala dysfunction (Walker et al., 2011). Our 7-month tgHD animals showed normal conditioning and extinction, which confirms their presymptomatic status. Along with presymptomatic alteration of prefronto-striatal processing in 5–6-month old tgHD animals (Höhn et al., 2011), we may suggest that part of the alteration of the impact of emotion on temporal processing might be due to prefronto-amygdala dysfunction even though this alteration did not impact the fear response or fear extinction. Interestingly, the difference in results between these two animal models in “emotion” extinction suggests that prefronto-amydgala alteration may not be the only factor. An alternative view may be that the medial central nucleus of amygdala is located in circuits functionally linking emotion learning (i.e., amygdala circuits) and interval timing learning (i.e., fronto-striatal circuits) (Buhusi and Meck, 2005; Matthews et al., 2012), allowing the fear-CS emotional value to influence temporal processing. In any case, our results highlight the fact that a modified interaction between emotion and executive function may be a sensitive marker of a presymptomatic status of HD in absence of altered emotional behaviors (i.e., conditioning and extinction). Further studies will be needed to tease apart its potential source (e.g., attention, decision) and underlying neural networks.

Previous results from our laboratory have shown that presymptomatic tgHD animals demonstrate decreased anxiety, and that early symptomatic animals exhibit emotional blunting and hypersensitivity to negative emotional situations along with a CE shrinkage (Faure et al., 2011). Similarly, emotion alteration seems to be manifold in HD patients, ranging from emotional blunting (depression and/or apathy) with a particular focus on negative emotion stimuli (Snowden et al., 2008; Hayes et al., 2009), to responses with dysphoric mood, including increases in anger, fear and arousal compared with healthy volunteers (Paradiso et al., 2008). The present results showing a modified time course of the modulation of temporal processing by emotion (reduced immediate, but delayed impact) extend this complex pattern in HD, and confirm that alteration of executive functions by emotion might be present at a presymptomatic stage. Moreover, convergence between the effect in tgHD models and medial CE lesioned animals, along with known neuropathology in this model, strongly suggest an early dysfunction of the central nucleus of amygdala in tgHD animals sustaining these disrupted emotional effects.

In conclusion, the present work demonstrates that emotion (fear) conditioned stimuli have an acute detrimental after-effect on executive function, as observed through a reduced temporal precision during a restricted 20 s period. Moreover, it indicates that the impact of emotion on time perception is altered in presymptomatic tgHD animals, which might be related to dysfunction of the central nucleus of amygdala. These results extend our knowledge about early dysfunctional circuits in HD emotional symptoms, and suggest that an alteration of their interaction with executive functions may be a sensitive presymptomatic marker of the emotional symptoms in animal models of HD.

### Conflict of interest statement

The authors declare that the research was conducted in the absence of any commercial or financial relationships that could be construed as a potential conflict of interest.
